# Plasmapheresis vs Conventional Insulin Therapy in Hypertriglyceridemia-Induced Acute Pancreatitis

**DOI:** 10.7759/cureus.40568

**Published:** 2023-06-17

**Authors:** Ijeoma Orabueze, Alicia Masucci, Valerie Cluzet

**Affiliations:** 1 Internal Medicine, Vassar Brothers Medical Center Nuvance Health, Poughkeepsie, USA; 2 Infectious Diseases, Vassar Brothers Medical Center Nuvance Health, Poughkeepsie, USA

**Keywords:** familial hypertriglyceridemia, insulin therapy, acute pancreatitis, hypertriglyceridemia, plasmapheresis

## Abstract

Hypertriglyceridemia is a rare yet firm etiology of pancreatitis, with an incidence of 2-4% in the general population. The etiology of hypertriglyceridemia itself consists of both primary and secondary causes. We discuss the case of a 37-year-old female with a strong family history of hypertriglyceridemia (primary cause) along with daily alcohol consumption (secondary cause) who initially presented to the emergency department with tingling and numbness of her bilateral upper extremities, bilateral lower extremity cramping and spasm and pins, and needles sensation in all extremities. She was found to have acute pancreatitis (AP) as a cause of hypocalcemia with elevated triglycerides of 5,823 mg/dl responsive to plasmapheresis combined with insulin drip. We explore the pathophysiology of hypertriglyceridemia-induced acute pancreatitis and the different modalities used to treat it which are still largely debated. The choice of therapy has been influenced by the cost, perceived effectiveness, and availability.

## Introduction

Acute pancreatitis is an inflammatory disease of the pancreas mostly caused by gallstones and alcohol use. It usually manifests as a sudden onset of severe epigastric pain which radiates to the back and can cause severe electrolyte abnormalities like hypocalcemia. Acute pancreatitis caused by hypertriglyceridemia consists of approximately 1-4% of the cases of hypertriglyceridemia. However, hypertriglyceridemia is considered more serious than other etiologies [1]. Hypertriglyceridemia usually leads to acute pancreatitis when the triglyceride levels are greater than 500 mg/dL. Usually, less than one in 5000 people have triglyceride levels greater than 1000 mg/dL, but our patient presented with hypertriglyceridemia of 5823 mg/dL [1]. To date, there is no designated first-line treatment in the management of hypertriglyceridemia-induced acute pancreatitis, with insulin therapy, insulin in combination with heparin, plasmapheresis or hemofiltration techniques, dietary modifications, and/or fibrates being the available options. Our patient received plasmapheresis which was found to be effective in preventing any complications of acute pancreatitis (AP) and rapidly reducing her triglyceride levels.

## Case presentation

A 37-year-old female presented to the emergency department (ED) with an acute onset of numbness of the upper extremities bilaterally, hand cramping, and inability to fully move her digits. She also noted a tingling sensation in both upper extremities which radiated down her hands. She developed cramping in bilateral lower extremities while at the ED but had no blurry vision, double vision, headache, or neck pain. She noted nausea, vomiting, abdominal pain, and diarrhea the week before after an episode of binge drinking vodka prior to the onset of symptoms but those resolved before she came to the emergency department. She stated that she did not seek medical attention because her symptoms had improved. Trousseau and Chvostek's signs were positive on presentation to the ED. However, she was found to have significant electrolyte abnormalities like hypokalemia of 3.6 mmol/l, hypomagnesemia of 1 mg/dl, hypocalcemia of 4.8 mg/dl, and hypophosphatemia of 1.4 mg/dl. She was also found to have a lactic acid level of 2 mmol/l (normal range 0.5-2.2 mmol/l), elevated lipase of 641 u/l, and severely elevated triglycerides of 5823 mg/dl. EKG done revealed sinus tachycardia with a prolonged QTC of 537 ms. 

**Figure 1:**
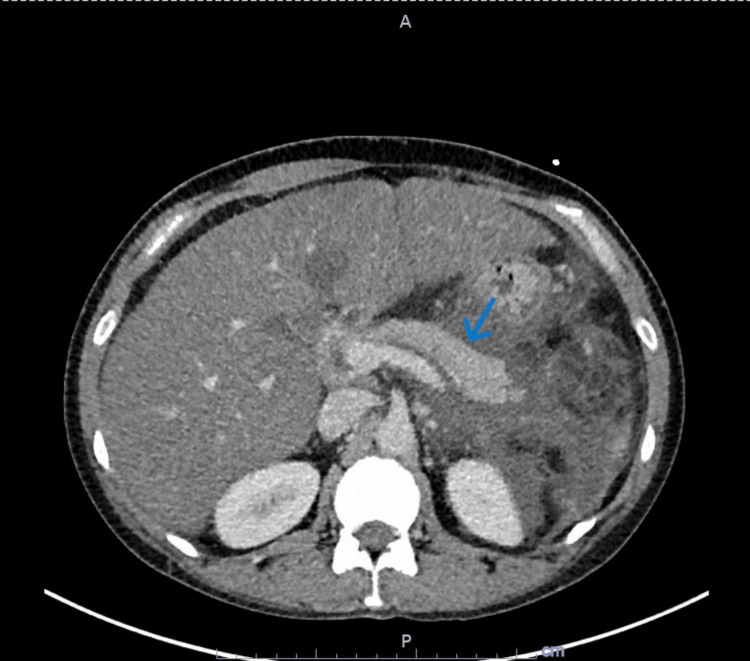
CT abdomen and pelvis with IV contrast Extensive fluid and fat stranding in the left abdomen, including in the lesser sac, which is compatible with clinically suspected pancreatitis. There is no loculated fluid collection identified. The pancreatic parenchyma is enhancing throughout without evidence of pancreatic necrosis. IV: Intravenous

**Figure 2:**
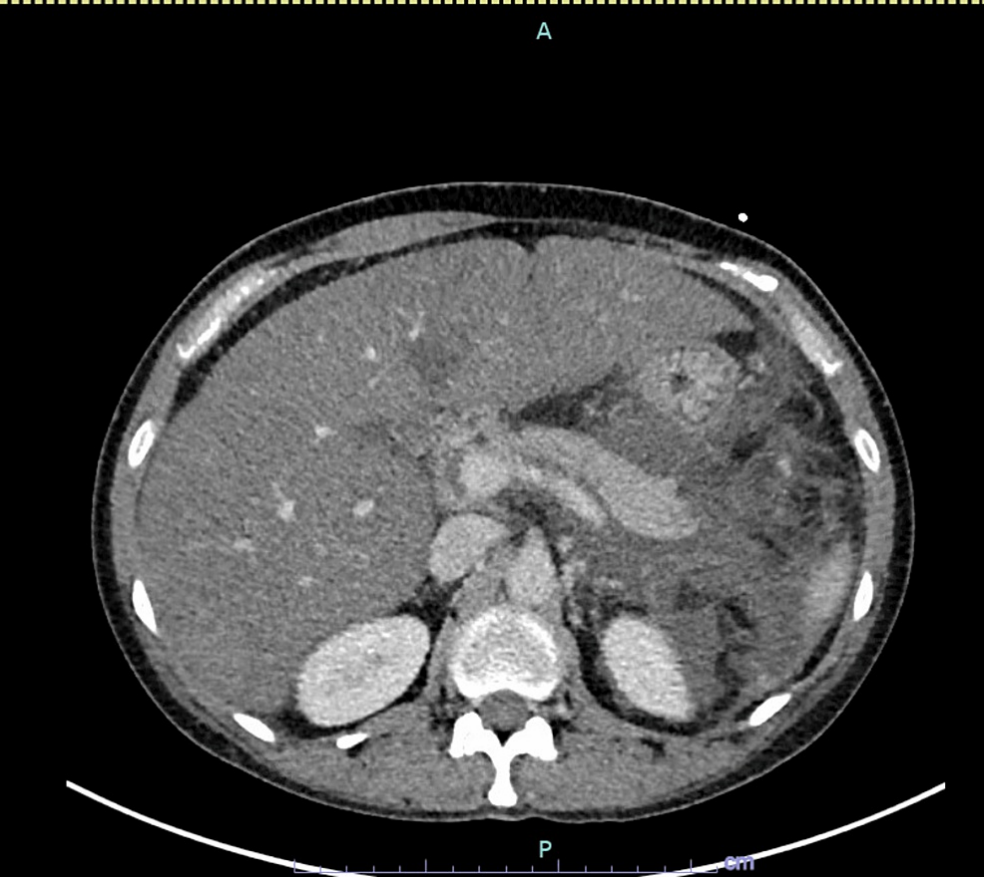
CT abdomen and pelvis with IV contrast Extensive induration of fat as well as free fluid in the left abdomen, including surrounding the pancreas, is compatible with acute pancreatitis. No loculated fluid collection or evidence of pancreatic parenchymal necrosis at this time. IV: Intravenous

CT abdomen and pelvis with intravenous (IV) contrast were ordered which revealed extensive induration of fat as well as free fluid in the left abdomen surrounding the pancreas compatible with acute pancreatitis (Figures 1-2). There was no parenchymal necrosis or loculated fluid collection. There was also hepatomegaly with low-density hepatic parenchyma representing fatty infiltration. She noted a family history of hypertriglyceridemia on her mother's side and hypercholesterolemia on her father's side. On presentation to the emergency department, she was febrile at 100.7 °F, tachycardic to 110 s, and hypertensive at 148/100 mmHg. Urine toxicology was negative and she had mild leukocytosis. She was initiated on 2.5 L of normal saline, calcium gluconate, magnesium sulfate, and K-Phos and admitted to the critical care unit.

Her electrolyte derangement was found to be multifactorial in the setting of acute pancreatitis, vomiting, and poor oral intake. Parathyroid hormone (PTH) and vitamin D levels were obtained. Vitamin D levels came back less than 5 ng/ml and PTH levels came back high at 161 pg/ml (15-65 pg/ml). She was started on an insulin drip with aggressive IV fluid resuscitation, a Foley catheter was inserted for input and output monitoring and her electrolytes were replenished accordingly. However, her triglyceride levels remained above 4000 mg/dl and the patient required plasmapheresis. With plasmapheresis, her triglyceride levels dropped to 1020 mg/dl and then 336 mg/dl. 

**Figure 3:**
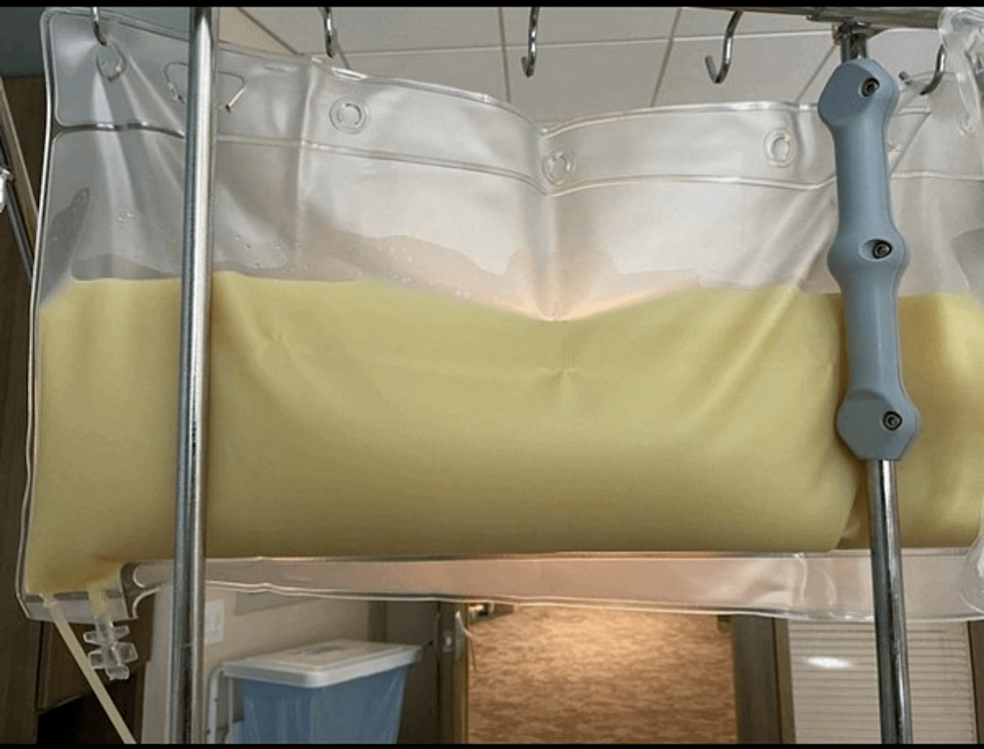
Lipid-rich fluid obtained after plasmapheresis

Below is a picture of the fluid taken from plasmapheresis (Figure 3). When appropriate, she was advanced to a clear liquid diet and then a low-fat diet. She was then started on atorvastatin 40 mg daily and vitamin D was repleted accordingly. She was discharged on the 6th day on fenofibrate, atorvastatin, and vascepa. She was managed in the intensive care unit for three days and downgraded soon after that.

## Discussion

Gallstones and alcohol abuse are the two most common causes of acute pancreatitis (AP). Hypertriglyceridemia (HTG) is a rare but well-established cause of acute pancreatitis, with an incidence of 2-4% [2]. It is thought to occur through hydrolysis of triglycerides (TG) by pancreatic lipase and excessive formation of free fatty acids with inflammatory changes, capillary injury, and hyperviscosity. Serum triglyceride levels above 1000 mg/dl (11.3 mmol/l) are usually considered necessary to ascribe causation for acute pancreatitis. However, this threshold is arbitrary, and the true level above which acute pancreatitis may occur is unknown [3]. This patient had a maternal history of hypertriglyceridemia and a history of alcohol consumption which both predispose to acute pancreatitis.

Patients with AP typically present with mid-epigastric and/or right upper quadrant pain that is constant, stabbing in character with radiation to the back. This patient did report abdominal pain that resolved before presentation, but worthy to note was her presentation with overt symptoms of hypocalcemia and other electrolyte derangements [4].

Different therapies in the management of HTG-induced acute pancreatitis include insulin therapy, insulin in combination with heparin, plasmapheresis or hemofiltration techniques, dietary modifications, and/or fibrates and Combined Blood Purification Therapy (CBPT) [2,5]. CBPT is a combination of plasmapheresis and continuous venovenous hemofiltration. It has been shown to improve 28-day mortality, but studies on this technique were severely limited [2]. Hence, there are no strong recommendations regarding its use in the management of HTG-induced acute pancreatitis [2]. Conventional therapy with insulin works by decreasing TG by promoting the synthesis and activity of lipoprotein lipase (LPL), which hydrolyzes TG into fatty acids and glycerol and facilitates the storage of the fatty acids in adipocytes [5]. This patient received an insulin drip with aggressive intravenous fluids and repletion of electrolytes, but triglyceride levels remained persistently elevated. Hence, she was transitioned to plasmapheresis. Triglyceride (TG) levels rapidly decreased after one session. Reducing serum TG levels quickly is crucial in the early treatment of HTG-AP [6]. Studies analyzing the effect of plasmapheresis for HTG-AP have been scarce with generally low sample sizes. However, several studies have shown that plasmapheresis is an effective tool for lowering TG levels [7].

In patients with worrisome features including lactic acidosis, hypocalcemia, organ dysfunction, and systemic inflammatory response syndrome (SIRS), plasmapheresis is a useful first-line therapy. In a retrospective study performed on three patients, the mean decrease in TG level was approximately 70% after one session, which correlates with the 82% drop seen in our patient thus showing that plasmapheresis is a useful tool for immediately decreasing TG to safe levels [7]. Diverse studies on the use of insulin therapy in HTG-induced AP have yielded inconsistent results, with some studies targeting euglycemia instead of triglyceride (TG) levels, uncertain dose and route of administration, and triglyceride lowering rates [5]. A previous case report showed persistently elevated TG levels (>2000 mg/dl) after 20 hours of insulin therapy and plasmapheresis (PEX) was initiated by our nephrologist with a rapid response after the first therapy to a level of 451 mg/dl, and 69 mg/dl following the second section [8]. PEX has been shown to rapidly remove TGs from circulation, preventing further pancreatic cellular injury. This occurs within hours, compared to other conventional therapy which takes days to achieve the same response, with a 50 to 80 % reduction after the first session. Therefore, it is highly recommended as initial therapy for extreme levels of TG with life-threatening symptoms. Furthermore, by removing proinflammatory markers and cytokines, the inflammatory process is downregulated, improving HTG-AP outcomes [2].

Combining statin therapy with fenofibrate has been shown to decrease TG levels considerably in comparison to monotherapy. The safety profile of combination versus monotherapy was comparable. Dietary modification and dietetics consultation should be strongly considered in addition to pharmacological therapy [7]. Some studies have noted that patients with HTG-AP TG levels > 5000 mg/dl who received plasmapheresis experienced shorter hospital courses and decreased hospital readmissions compared to patients who did not receive plasmapheresis. However, no evidence currently exists to support the effect on mortality reduction [7]. Our patient had TG levels higher than 5000 mg/dl and we argue that plasmapheresis was the most effective therapy for her. In Figure 3 above, of the plasma filtered from our patient’s blood, we can see the degree of lipemia, and she could have been at risk of more severe complications like pancreatic necrosis and multiorgan dysfunction. 

## Conclusions

Although there is no conventional first-line therapy in the management of HTG-induced AP, plasmapheresis is an effective treatment for patients presenting with severe HTG-induced AP who are at risk of complications and should be encouraged as initial therapy. More studies are needed to further elucidate this treatment modality, weighing the risk and benefits.
